# RETRACTION: Mental Health Condition of Caregivers Affected Metal Health of Burn Injury Patients

**DOI:** 10.1111/iwj.70553

**Published:** 2025-04-18

## Abstract

**RETRACTION:** QinB. , LiX. , ZhangH. , ZhangC. , and ZhaoL. , “Mental Health Condition of Caregivers Affected Metal Health of Burn Injury Patients,” International Wound Journal 20, no. 5 (2023): 1376–1383, 10.1111/iwj.14000.36718494 PMC10088817

The above article, published online on 08 November 2022, in Wiley Online Library (http://onlinelibrary.wiley.com/), has been retracted by agreement between the journal Editor in Chief, Professor Keith Harding; and John Wiley & Sons Ltd. Following an investigation by the publisher, all parties have concluded that this article was accepted solely on the basis of a compromised peer review process. The editors have therefore decided to retract the article. The authors did not respond to our notice regarding the retraction.

**RETRACTION:** B. Qin , X. Li , H. Zhang , C. Zhang , and L. Zhao , “Mental Health Condition of Caregivers Affected Metal Health of Burn Injury Patients,” International Wound Journal 20, no. 5 (2023): 1376–1383, 10.1111/iwj.14000.36718494 PMC10088817

The above article, published online on 08 November 2022, in Wiley Online Library (http://onlinelibrary.wiley.com/), has been retracted by agreement between the journal Editor in Chief, Professor Keith Harding; and John Wiley & Sons Ltd. Following an investigation by the publisher, all parties have concluded that this article was accepted solely on the basis of a compromised peer review process. The editors have therefore decided to retract the article. The authors did not respond to our notice regarding the retraction.

